# Complicated Japanese Spotted Fever With Meningitis in an Older Patient: A Case Report

**DOI:** 10.7759/cureus.50681

**Published:** 2023-12-17

**Authors:** Shiho Amano, Shinichiro Suyama, Nozomi Nishikura, Chiaki Sano, Ryuichi Ohta

**Affiliations:** 1 Community Care, Unnan City Hospital, Unnan, JPN; 2 Internal Medicine, Heisei Memorial Hospital, Unnan, JPN; 3 Community Medicine Management, Shimane University Faculty of Medicine, Izumo, JPN

**Keywords:** rash variability, comprehensive management, serological testing, diagnostic challenges, aseptic meningitis, bacteremia, atypical presentation, older patients, tick-borne infections, japanese spotted fever

## Abstract

Japanese spotted fever (JSF) poses a significant public health challenge, mainly due to its atypical presentation in specific demographics. This report details a unique case of JSF in an 89-year-old female who was admitted to a rural hospital exhibiting generalized pain and rapid cognitive decline but no rash. Initially misdiagnosed as polymyalgia rheumatica, her condition was complicated by thrombocytopenia and altered mental state, prompting consideration of tick-borne illnesses. Subsequent serological analysis confirmed JSF despite the absence of its hallmark rash. The patient's condition escalated to include bacteremia and aseptic meningitis. Treatment involved a regimen of minocycline and meropenem, along with endoscopic cauterization of a bleeding rectal ulcer. After treatment, the patient showed improvement and was transferred for rehabilitation. This case highlights the criticality of considering JSF in elderly patients within endemic areas, even when classic symptoms like erythema and petechiae are absent. It underscores the necessity for broad diagnostic perspectives, especially in atypical presentations, and the integration of comprehensive care approaches. The involvement of caregivers and relatives in early detection and seeking medical care promptly is crucial. The report illustrates the complexities in diagnosing and managing advanced JSF cases and stresses the importance of early serological testing and adaptive treatment strategies in managing such challenging cases.

## Introduction

Japanese spotted fever (JSF), a significant health concern caused by *Rickettsia japonica* transmitted through tick bites [1], presents a complex clinical picture that challenges timely diagnosis and management. Commonly manifested symptoms include fever, systemic muscular pain, fatigue, appetite loss, and joint pain [2]. Notably, the development of rashes, such as erythema and petechiae, is a crucial diagnostic indicator for JSF, aiding in the early initiation of effective treatment strategies to improve patient outcomes [2]. However, the unpredictable onset of these rashes often results in diagnostic delays, complicating the clinical management of the disease [1,3].

The variability in rash appearance, a key diagnostic marker for JSF, introduces significant challenges in accurately identifying the disease, especially in atypical presentations [4]. This unpredictable nature of symptom manifestation underscores the importance of considering alternative diagnostic approaches, particularly in cases where the rash is delayed or absent [4].

In this case report, we examine the diagnostic journey of an elderly woman who presented with rapid cognitive decline and polyarticular pain, indicative of JSF, but without the typical rash [1,4]. This case, marked by its diagnostic intricacies, highlights the need for heightened vigilance and a comprehensive approach to JSF diagnosis, particularly in patients showing atypical symptomatology. Our discussion delves into the challenges faced, the diagnostic strategies employed, and the broader implications for JSF management, emphasizing the importance of an adaptable and thorough approach in the face of such diagnostic complexities [2-4]. This report aims to contribute to the growing body of knowledge on JSF, providing insights into managing the disease when confronted with non-standard presentations.

## Case presentation

An 89-year-old woman, previously independent in daily activities and living with her family, presented to a rural community hospital with generalized pain. Her recent history included one week of fatigue and frequent falls starting two days before admission. Initially, she sought care at the orthopedic department due to polyarticular and muscular pain that hindered her daily functions, such as meal preparation and self-care. Her medical history comprised hypertension, dyslipidemia, and bilateral knee osteoarthritis, with a long-term prescription of amlodipine (5 mg). The choice of amlodipine, a calcium channel blocker, was due to its effectiveness in managing her hypertension with minimal side effects, given her age and comorbidities.

Upon admission, her vital signs were notable for a mild fever (37.3 ℃), tachycardia (96 beats/min), and hypotension (106/65 mmHg). Her mental status was altered, not being alert to time, place, or person. Physical examination showed muscle tenderness in the proximal limbs but no evident movement disorder, skin rashes, joint inflammation, or temporal artery abnormalities. The initial laboratory tests revealed thrombocytopenia, elevated liver enzymes, hyperferritinemia, and raised C-reactive protein levels, suggesting an acute inflammatory process (Table 1).

**Table 1:** Initial laboratory data of the patient eGFR, estimated glomerular filtration rate; CRP, C-reactive protein; MPO-ANCA, myeloperoxidase antineutrophil cytoplasmic antibody.

Parameter	Level	Reference
White blood cells	5.9	3.5-9.1 × 10^3^/μL
Red blood cells	3.87	3.76-5.50 × 10^6^/μL
Hemoglobin	11.5	11.3-15.2 g/dL
Hematocrit	35.3	33.4-44.9%
Mean corpuscular volume	91.2	79.0-100.0 fL
Platelets	7.2	13.0-36.9 × 10^4^/μL
Total protein	6.4	6.5-8.3 g/dL
Albumin	3.0	3.8-5.3 g/dL
Total bilirubin	0.7	0.2-1.2 mg/dL
Aspartate aminotransferase	145	8-38 IU/L
Alanine aminotransferase	56	4-43 IU/L
Alkaline phosphatase	90	106-322 U/L
γ-Glutamyl transpeptidase	19	<48 IU/L
Lactate dehydrogenase	420	121-245 U/L
Blood urea nitrogen	34.0	8-20 mg/dL
Creatinine	0.60	0.40-1.10 mg/dL
eGFR	90.0	>60.0 mL/min/L
Serum Na	132	135-150 mEq/L
Serum K	3.8	3.5-5.3 mEq/L
Serum Cl	96	98-110 mEq/L
CRP	11.32	<0.30 mg/dL
Ferritin	2092	3.6-114 ng/mL
Anti-nuclear antibody	40	<40
MPO-ANCA	<1.0	<3.5 U/mL
Urine test		
Leukocyte	Negative	Negative
Nitrite	Negative	Negative
Protein	Negative	Negative
Glucose	Negative	Negative
Bilirubin	Negative	Negative
Blood	Negative	Negative
pH	7.0	
Specific gravity	1.013	

These findings directed the initial clinical suspicion toward a systemic inflammatory response, potentially secondary to an infectious or autoimmune cause.

Given her age, symptoms, and initial findings, a diagnosis of polymyalgia rheumatica was considered, and prednisone (10 mg) was initiated. Prednisone, a corticosteroid, was chosen for its potent anti-inflammatory properties, which are beneficial in treating polymyalgia rheumatica. However, her condition was complicated with persistent thrombocytopenia and altered mental status, prompting a reevaluation of the diagnosis. Notably, the region's high incidence of tick-related infections and the absence of typical rash or tick bite marks initially masked the consideration of JSF.

Serological tests for JSF, tsutsugamushi disease, and severe fever with thrombocytopenia syndrome were performed due to the geographical context and the clinical picture of fever, thrombocytopenia, and acute onset of neurological symptoms. Concurrently, minocycline (200 mg/day) was started intravenously, anticipating a possible tick-borne infection. Minocycline, a tetracycline antibiotic, was selected for its efficacy against a broad range of bacteria, including Rickettsia species, and its ability to penetrate the central nervous system, which was crucial given her neurological symptoms.

The patient's progression to acute unconsciousness, tachycardia, and abdominal tenderness raised additional concerns, leading to an abdominal CT scan. The scan did not show abscess formation or free air but suggested bacterial translocation, for which meropenem (3 g/day) was initiated. Meropenem, a broad-spectrum carbapenem antibiotic, was chosen for its effectiveness against a wide range of bacterial pathogens, particularly in cases of suspected bacterial translocation and sepsis.

**Figure 1:**
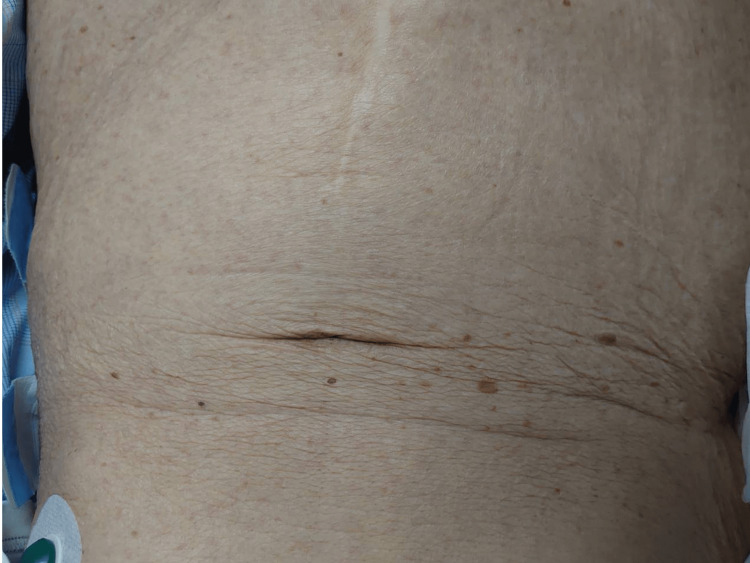
The appearance of erythema on the abdomen was sparse

On the sixth day, symptoms of neck stiffness and generalized rigidity directed the clinical suspicion toward meningitis. CSF analysis showed elevated protein levels but normal cell count and no bacterial growth, indicative of aseptic meningitis. On the same day, erythema appeared over the entire body, including the palms (Figure 1).

**Figure 2:**
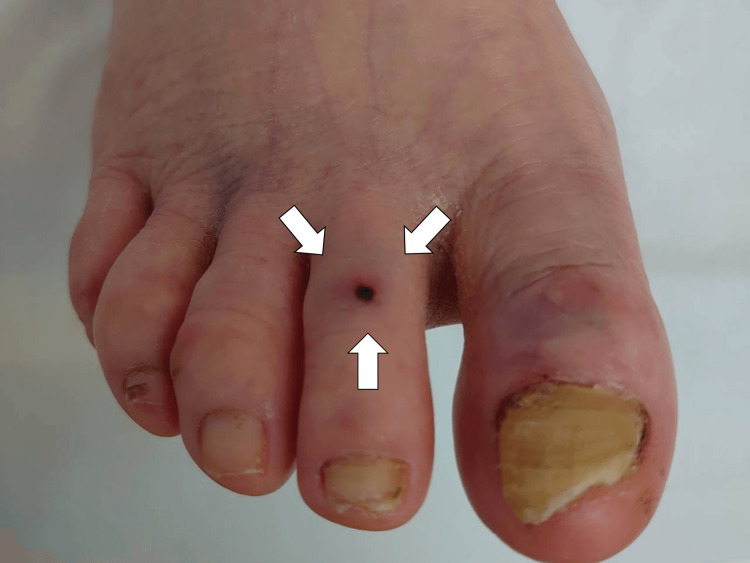
The wound of tick bite on the right foot (white arrows)

A critical reexamination of her skin on day 6 revealed a tick bite mark on her right foot's second toe, a vital clue that had been initially overlooked (Figure 2).

This finding and the positive serological tests for *Rickettsia japonica* DNA and immunoglobulins M and G on the same day confirmed the diagnosis of JSF with aseptic meningitis. The presence of Rickettsia japonica DNA in the serological tests was significant as it directly indicated the presence of the causative agent of JSF. The DNA was identified using polymerase chain reaction (PCR) techniques, which are highly specific and sensitive for detecting Rickettsial DNA. The detection of immunoglobulins M and G was also crucial. Immunoglobulin M (IgM) antibodies typically indicate recent or acute infection. In contrast, immunoglobulin G (IgG) antibodies suggest either a past infection or a more progressed stage of the current infection. In conjunction with the patient's symptoms and the PCR results, the simultaneous detection of both IgM and IgG antibodies strongly supported the diagnosis of an active JSF infection. These serological markers, the clinical presentation, and the discovery of the tick bite provided a robust basis for confirming JSF as the underlying cause of the patient's symptoms.

The patient's management included continued intravenous minocycline treatment, leading to gradual improvement in her fever, liver function, and thrombocytopenia. The ongoing use of minocycline was sustained due to its effectiveness in addressing the underlying Rickettsial infection. However, on the 19th day, she developed bloody stools, and a rectal ulcer was discovered, requiring endoscopic cauterization and blood transfusions. Her subsequent gastrointestinal bacterial translocation due to *Citrobacter koseri* was managed with tazobactam/piperacillin. Tazobactam/piperacillin, a combination antibiotic, was introduced to counter the broad-spectrum gastrointestinal infection, likely secondary to the initial infection and treatment. Ultimately, the patient's condition stabilized, enabling her to transfer to the rehabilitation unit in preparation for home discharge.

## Discussion

This case report underscores a complex scenario where JSF manifested rapidly and severely in an elderly patient, uniquely absent of the typical rash and further complicated by bacteremia and meningitis. It illustrates the variability in JSF presentations, emphasizing the need for multifaceted management approaches, especially in older patients with fever of unknown origin (FUO) and other nonspecific symptoms.

The rapid and severe progression of JSF in this elderly patient, notably without the hallmark rash, calls for heightened vigilance among clinicians. A high suspicion of JSF is essential in such cases, even in the absence of classic symptoms, particularly in areas where JSF is prevalent [5,6]. The aged population often exhibits some degree of immune compromise, increasing their vulnerability to severe and uncharacteristic manifestations of infections like JSF [7]. This situation necessitates a comprehensive approach to care, addressing JSF and its direct complications, such as bacteremia and meningitis, and providing supportive care for the general frailty commonly observed in the elderly.

In instances of FUO, the possibility of JSF might not be immediately apparent, especially given the potential delay in rash appearance, typically five to seven days after disease onset. Clinicians should consider various differential diagnoses, including JSF, and prioritize early serological testing for timely and effective intervention [8,9]. In JSF patients, altered mental state and tachycardia should promptly consider concurrent infections, such as meningitis or systemic bacterial infections [10,11]. Prompt recognition and management of these complications by general physicians are crucial in averting critical outcomes [12]. The absence of rash as a guiding symptom in JSF diagnosis presents a unique challenge, emphasizing the importance of a thorough patient history, including exposure to endemic areas, and the utilization of advanced diagnostic methods to initiate appropriate treatment strategies.

Furthermore, to prevent acute exacerbation of JSF, more public education regarding JSF risks, presentation, and need for medical care is vital, particularly for older patients with JSF. Educating them about the disease's severity, potential complications, and care requirements can facilitate early detection and compliance with treatment protocols, especially in managing concurrent infections [13,14]. Early detection of the infection and prompt treatments are essential for quick recovery, addressing post-infection sequelae, and mitigating the chronic impacts of systemic bacterial infections [15]. In this case, the delay in seeking medical care might cause the deterioration of JSF and various complications. If the patient and their families had the knowledge of JSF, the patient could have had come to medical institutions earlier. To prevent deterioration, the comprehensive approach, including public information provision and education from general medicine, ensures early detection of JSF from various people such as patients, families, and friends, underscoring the role of general physicians in patient continual care [16]. This case provides valuable insights into the diverse and potentially severe presentations of JSF in the elderly, accentuating the importance of a broad diagnostic perspective, thorough management strategies, and vigilant long-term care.

## Conclusions

This case report illuminates the intricacies and heightened severity that JSF can exhibit in elderly patients, especially in those presenting atypically without the characteristic rash and with concurrent serious infections like bacteremia and meningitis. It underscores clinicians' need for increased vigilance for JSF in patients, irrespective of classic symptoms, particularly in endemic areas. A thorough approach incorporating detailed patient histories, prompt serological testing, and comprehensive management plans is vital for accurate diagnosis and effective treatment. Moreover, the importance of engaging healthcare professionals and family members in understanding the nuances of JSF's atypical manifestations cannot be overstated. Providing education about these less common presentations and ensuring early detection of the infection are crucial steps in ensuring optimal patient outcomes. Such collaborative and informed approaches are vital in managing the immediate effects of the infection. This case serves as a reminder of the diverse and severe nature of JSF presentations in older individuals, highlighting the imperative for a broad, informed, and proactive approach to patient care in such complex scenarios.
